# R5-SHIV Induces Multiple Defects in T Cell Function during Early Infection of Rhesus Macaques Including Accumulation of T Reg Cells in Lymph Nodes

**DOI:** 10.1371/journal.pone.0018465

**Published:** 2011-04-05

**Authors:** Michael Santosuosso, Elda Righi, E. David Hill, Pierre R. Leblanc, Brett Kodish, Hari N. Mylvaganam, Nagadenahalli B. Siddappa, Liljana Stevceva, Shiu-Lok Hu, Musie Ghebremichael, Agnes-L. Chenine, Avi-Hai Hovav, Ruth M. Ruprecht, Mark C. Poznansky

**Affiliations:** 1 Infectious Diseases Medicine Division, Vaccine and Immunotherapy Center, Massachusetts General Hospital, Charlestown, Massachusetts, United States of America; 2 Department of Cancer Immunology and AIDS, Dana-Farber Cancer Institute, Boston, Massachusetts, United States of America; 3 Department of Pharmaceutics, University of Washington, Seattle, Washington, United States of America; 4 Department of Biostatistics and Computational Biology, Harvard School of Public Health and Dana Farber Cancer Institute, Boston, Massachusetts, United States of America; 5 Faculty of Dental Medicine, Institute of Dental Sciences, Hebrew University, Jerusalem, Israel; New York University, United States of America

## Abstract

**Background:**

HIV-1 is a pathogen that T cell responses fail to control. HIV-1gp120 is the surface viral envelope glycoprotein that interacts with CD4 T cells and mediates entry. HIV-1gp120 has been implicated in immune dysregulatory functions that may limit anti-HIV antigen-specific T cell responses. We hypothesized that in the context of early SHIV infection, immune dysregulation of antigen-specific T-effector cell and regulatory functions would be detectable and that these would be associated or correlated with measurable concentrations of HIV-1gp120 in lymphoid tissues.

**Methods:**

Rhesus macaques were intravaginally inoculated with a Clade C CCR5-tropic simian-human immunodeficiency virus, SHIV-1157ipd3N4. HIV-1gp120 levels, antigen-specificity, levels of apoptosis/anergy and frequency and function of Tregs were examined in lymph node and blood derived T cells at 5 and 12 weeks post inoculation.

**Results/Conclusions:**

We observed reduced responses to Gag in CD4 and gp120 in CD8 lymph node-derived T cells compared to the peripheral blood at 5 weeks post-inoculation. Reduced antigen-specific responses were associated with higher levels of PD-1 on lymph node-derived CD4 T cells as compared to peripheral blood and uninfected lymph node-derived CD4 T cells. Lymph nodes contained increased numbers of Tregs as compared to peripheral blood, which positively correlated with gp120 levels; T regulatory cell depletion restored CD8 T cell responses to Gag but not to gp120. HIV gp120 was also able to induce T regulatory cell chemotaxis in a dose-dependent, CCR5-mediated manner. These studies contribute to our broader understanding of the ways in which HIV-1 dysregulates T cell function and localization during early infection.

## Introduction

It is increasingly clear that a single HIV virion or a small founder virus population infects a target cell in the mucosa and initiates a localized infection that is followed by systemic spread of the virus [1], [2], [3], [4]. Early HIV infection is characterized by a reduction of CD4 T cells approximately 9 days post onset of acute illness, followed by an increased number of CD8 T cells and an inversion of the CD4/CD8 T cell ratio [5], [6], [7]. These kinetics are associated with an increasing plasma viral load and rising numbers of HIV-specific CD8 T cells. By 3–6 months post-inoculation, the plasma viral load has equilibrated to a set point that is highly correlated with disease progression [5], [8], [9]. These early events set the stage for a prolonged and multi-dimensional negative impact on the host immune system that characteristically fails to eradicate the infection [8], [9], [10], [11].

HIV proteins, including the surface envelope glycoprotein gp120, perform critical functions during the viral life cycle as well as playing a direct role in the immune pathogenesis of HIV/AIDS. For example, it has been shown that HIV-1 gp120 can mediate both viral entry and dysregulate immune cell function through its well-described interaction with cellular receptors, including CD4 and the chemokine co-receptors, CXCR4 and CCR5 (for X4 and R5 tropic viruses, respectively). HIV gp120 has been extensively studied *in vitro* with respect to its effects on a variety of both stromal and immune cell types [10], [11], [12]. HIV gp120 can impair dendritic cell maturation or induce dendritic cells to become immunosuppressive [12], [13], [14]. HIV gp120 has been shown *in vitro* to dysregulate T cell functions, including TCR desensitization, interference with co-stimulation, induction of apoptosis, and T cell migration [15], [16], [17], [18], [19], [20], [21]. The impairment of these functions may have a dramatic effect on the formation and stability of effective anti-HIV immunity beyond the characteristic depletion of CD4 T cells seen in HIV/AIDS.

*In vivo*, gp120-induced immune dysfunction is more difficult to quantify, although human studies have consistently shown that gp120 is poorly targeted by both humoral and cellular immune responses [22], [23], [24], [25]. In addition, there is a growing body of evidence to support the view that levels of gp120 required for immune dysregulation *in vitro* are present in lymphoid tissues *in vivo* [26]. Accumulation of high levels of gp120 in lymphoid organs of early SHIV-infected rhesus monkeys (RM) have been demonstrated [27], and high levels of the envelope protein have been found in the lymphoid tissues of HIV-1-infected humans with very low or undetectable viral loads [28], [29]. These data support the view that the earliest events after primary infection, including exposure of immune cells to high local or systemic levels of gp120, play a major role in the long-term outcome of infection [1], [3], [30]. Taken together, these studies support the view that HIV-1 gp120 may play a role in immune dysfunction in lymphoid tissues as infection progresses.

We examined the hypothesis that a biologically relevant R5 HIV-1 gp120, in the context of early mucosal SHIV challenge, results in multimodal dysregulation of T cell-mediated immune function that in some cases, are associated with persistently high levels of gp120 in lymphoid tissues. Mucosal R5-SHIV inoculation of RM was chosen as a model system because 90 percent of all HIV infections among humans involve mucosal exposure to an R5-tropic virus. In this non-human primate study, we demonstrate early immune dysregulation of CD8 gp120-specific and CD4 Gag-specific T cells in the lymph nodes (LN), as compared to the peripheral blood. We found no enhancement of antigen-induced cell death in LN derived T cells compared to the T cells from the peripheral blood (PB); however, we observed increased levels of PD-1 expression on CD4 T cells in the LN as compared to PB and naïve LN samples. Furthermore, LN gp120 levels correlated with an increased proportion of regulatory T cells (Treg) in lymphoid tissues. Depletion of CD4^+^CD25^+^ Treg cells augmented Gag-specific CD8 T cell responses, whereas gp120-specific T cells remained impaired in this context. Finally, we demonstrated that R5 gp120 could induce Treg chemotaxis in vitro in a CCR5 mediated and concentration dependent manner. This study demonstrates multiple T cell-mediated immune defects that are associated with HIV-1 gp120 in LN during the first 12 weeks of R5 clade C SHIV infection.

## Materials and Methods

### Ethics Statement

The studies were conducted in accordance with National Institute of Health guidelines on the care and use of laboratory animals at the Yerkes National Primate Research Center ((YNPRC), Emory University, Atlanta, GA), which is fully accredited by the Association for Assessment and Accreditation of Laboratory Animal Care International. The Animal Care and Use Committee of YNPRC and DFCI approved all animal experiments.

### Animals

Six rhesus macaques (RM) of Indian origin were exposed intravaginally to various dilutions of a SHIV-1157ipd3N4 (R5-SHIV) stock grown in RM peripheral blood mononuclear cells (PBMC) (227 ng/ml of p24; 4×10^6^ /ml) 50% tissue culture infectious doses (TCID_50_) as titrated in TZM-bl cells. This virus is exclusively R5-tropic [31], [32]. Two animals that received the lowest mucosal dose (1∶50) of SHIV did not seroconvert and were challenged secondarily with 1 ml of viral stock via the intravenous route (Table 1). Peripheral blood (PB) and peripheral nodes, including axillary LN were sampled at 5 weeks and 12 weeks post-inoculation. Some LN were frozen and stored at -80°C for later quantitation of gp120. Mononuclear cells (MNC) were isolated from PB via Ficoll gradient enrichment as previously described [27]. MNC were obtained from the LN via physical disruption with cell lifters in a Petri dish and enriched by Ficoll gradient centrifugation. Samples were frozen at −80°C prior to use *in vitro* assays.

**Table 1:** Levels of gp120 and p27 in the Plasma, Lymph Node and Spleen of RM 12 weeks post-inoculation with R5-SHIV.

Animal	Tissue	gp120 (pg/ml)	p27 (pg/ml)
Rbo-6	Plasma	0.00	21.53
	LN	71.81	1832.04
	Spleen	297.63	545.57
RBw-8	Plasma	0.00	62.82
	LN	170.37	447.72
	Spleen	278.16	603.89
Ryb-6	Plasma	0.00	7.54
	LN	176.96	687.00
	Spleen	451.38	1064.68

LN: Lymph Node.

### Quantitation of HIV gp120 and SIV p27 in tissue

LN biopsy specimens from infected and non-infected animals were thawed, weighed and suspended in 3.5 ml of lysis buffer (RIPA containing protease inhibitors), per gram. After being subjected to two freeze/thaw cycles, samples were spun at 16,000× g for 10 min and supernatants were frozen at −80°C prior to analysis as previously described [29]. Thawed samples were diluted in RPMI1640 containing 10% fetal bovine serum (FBS) and aliquots were assayed using the HIV-1 gp120 Antigen Capture Assay (Advanced BioScience Laboratories, Inc., Kensington, MD) and a signal amplification kit ELAST ELISA Amplification (Perkin Elmer, San Jose, CA). Briefly, the modifications to the assay consisted of diluting the HIV-1 gp120 Antigen Capture Assay kit conjugate 1∶1 with 1% BSA PBS-Tween 20 (0.05%) followed by 60 min incubation at 37°C. Next, 100 µl of Biotinyl Tyramide Solution (ELAST ELISA Amplification) was added to each well, followed by a 20 min incubation at room temperature. Detection consisted of adding 100 µl of diluted Streptavidin–HRP Concentrate from the ELAST ELISA Amplification kit; 1000 fold with 1% BSA PBS-Tween 20 (0.05%) to each well and incubating the plate at room temperature for 30 min. The peroxidase substrate and the stop solution from the HIV-1 gp120 Antigen Capture Assay kit were used as described by the manufacturer. After stopping the reaction, absorption was measured at 450 nm as previously described [29]. SIV p27 was measured via a Retrotek SIV p27 antigen ELISA as per manufacturer's instructions (Zeptometrix, Buffalo, NY.). Antigen concentrations were calculated as the number picograms per ml of tissue, according to the following equation: concentration of antigen  =  {[(tissue volume + media added)/tissue volume] × antigen amount}/tissue volume.

### Flow cytometry and intracellular cytokine staining (ICS)

PB and LN derived MNC were stimulated for 5 hours in the presence of an inhibitor of Golgi function (Golgi plug, BD Biosciences, San Jose, CA) and anti-CD28 and anti-CD49d (clone CD28.2 and 9F10, BD Biosciences). Antigen-specific stimulation utilized 2 µg/ml of overlapping peptide pools from either a consensus gp120 of HIV clade C (kindly provided by Dr. Christian Brander), or from SIVmac239 Gag (AIDS Research and Reference Reagent Program, Division of AIDS, NIAID, NIH, 15mers). Multi-parameter surface staining for CD3, CD4, CD8, CD25, CD127, (CD3 clone Sp34-2, CD4 clone L200, CD8 clone SK1, CD25 clone M-A251, CD127 clone hIL-7R-M21 BD Biosciences) and PD-1 (clone EH12.2H7, Biolegend, San Diego, CA) were carried out, followed by permeabilization and intracellular staining for IFN-γ or FoxP3 as per manufacturer's instructions (IFN-γ clone B27 BD Biosciences, FoxP3 clone PHC101 eBiosciences). Analysis was completed using FlowJo Software (TreeStar, Ashland, OR) and gating was completed using a ‘fluorescence minus one’ (FMO) approach.

### Activation- Induced Cell Death (AICD)

A 96 well round-bottom plate was coated with 1 µg of anti-CD3 monoclonal antibody (clone Sp34-2, BD Biosciences) overnight at 4°C. Previously frozen samples from PB and LN were cultured in the presence or absence of plate-bound anti-CD3 with the addition of anti-CD28, anti-CD49d for 5 hours at 37°C and 5% CO_2_.

### CD25 Depletion and CD4+CD25+ Isolation

PB and LN derived MNC were depleted via a MACS CD25 depletion kit (Miltenyi Biotec, Auburn, CA) as per the manufacturer's instructions. CD25 depletion was confirmed via flow cytometry and was 78±8%. Depleted or non-depleted cells were then stimulated and stained as described above to examine the role of CD25 cells on T-effector cell functions. For migration studies, human PBMC were obtained via a Ficoll spin and CD4+CD25+ T regulatory cells were isolated via a Human CD4+CD25+ MACS isolation kit (Miltenyi Biotec, Auburn, CA), as previously described [27]. Cell subpopulation purity was confirmed via flow cytometry to be greater than 80%.

### Transmigration Assay

T cell migration was measured using Transwells (96-well format, 3-mm pore; ChemoTx System, Neuro Probe Inch, Gaithersburg, MA) as previously described [33]. Briefly, 7,000 CD4+CD25+ T-regs were loaded into the upper chamber, and 30 µl of medium alone or media supplemented with CCR5 tropic YU2 gp120 (ImmunoDiagnositcs, Woburn, MA) was added to the lower or upper chamber at the concentrations of 500 pg/ml, 5 ng/ml, and 500 ng/ml [34]. After a three hour incubation at 37°C and 5% CO2, cells in the upper chamber of the transwell were removed and migrated cells in the lower chamber were counted. using a hemocytometer. The normalized transmigration index was calculated as the ratio between the cells counted in the presence of R5 gp120 and when cells were exposed to media alone in upper and lower chambers. To determine whether T-cell migration was G protein-mediated, the cells were pre-incubated with 100 ng/ml pertussis toxin (PTx; Sigma Aldrich) for 1 h at 37°C, washed, and then loaded in the chemotaxis chamber. In order to determine whether the migration of cells in response to R5 gp120 gradients was CCR5 dependent, cells were pre-incubated with the CCR5 antagonist, TAK-779, at a concentration of 40 nM [34].

### Statistical analysis

All statistical analyses were performed in collaboration with Dr. Gebremichael at the Department of Biostatistics and Computational Biology at the Harvard School of Public Health. The analyses were completed using the Student *t*-Test and determinations of correlation coefficients using Excel software.

## Results

### Quantifiable amounts of gp120 in secondary lymphoid organs of RM at 12 weeks post mucosal R5-SHIV inoculation

We have previously demonstrated that gp120 is found in the secondary lymphoid organs of individuals with chronic HIV infection at levels that were disproportionally high in comparison to both local p24 concentrations or plasma viral loads[29]. We sought to examine if during early mucosal R5-SHIV challenge, R5 gp120 would also be found at high levels in LN and spleen. At 12 weeks post-inoculation, we observed measureable amounts of gp120 in LN as compared to PB (Table 1, 139.7±34 pg/ml). Interestingly, at 12 weeks, we also observed significantly more gp120 in the spleen of infected RM than in the LN (342.4±54.7 pg/ml, vs 12 week LN; p<0.05 non-paired T-test), in spite of the fact that half of the animals (3/6) had non-detectable viral loads in their PB (<1,300 copies/ml)[35]. Furthermore, we observed considerably lower levels of p27 than the expected Gag∶gp120 ratio would lead one to predict. Previous studies of the protein content of HIV virions have demonstrated a ratio of p24 to gp120 to vary between 6∶1 and 60∶1 (table 1, 12 weeks p<0.05 spleen, p<0.001 LN vs an expected 6∶1 Gag∶gp120 ratio, p<0.0001 60∶1 Gag∶gp120 ratio) [29], [36]. These data demonstrate that early after inoculation there is a gradient of gp120 between high detectable levels in LN, as described above, and undetectable amounts in PB at 12 weeks.

### Reduced Gag-specific CD4 and gp120 specific CD8 T cell responses in the LN of RM during early infection

**Figure 1:**
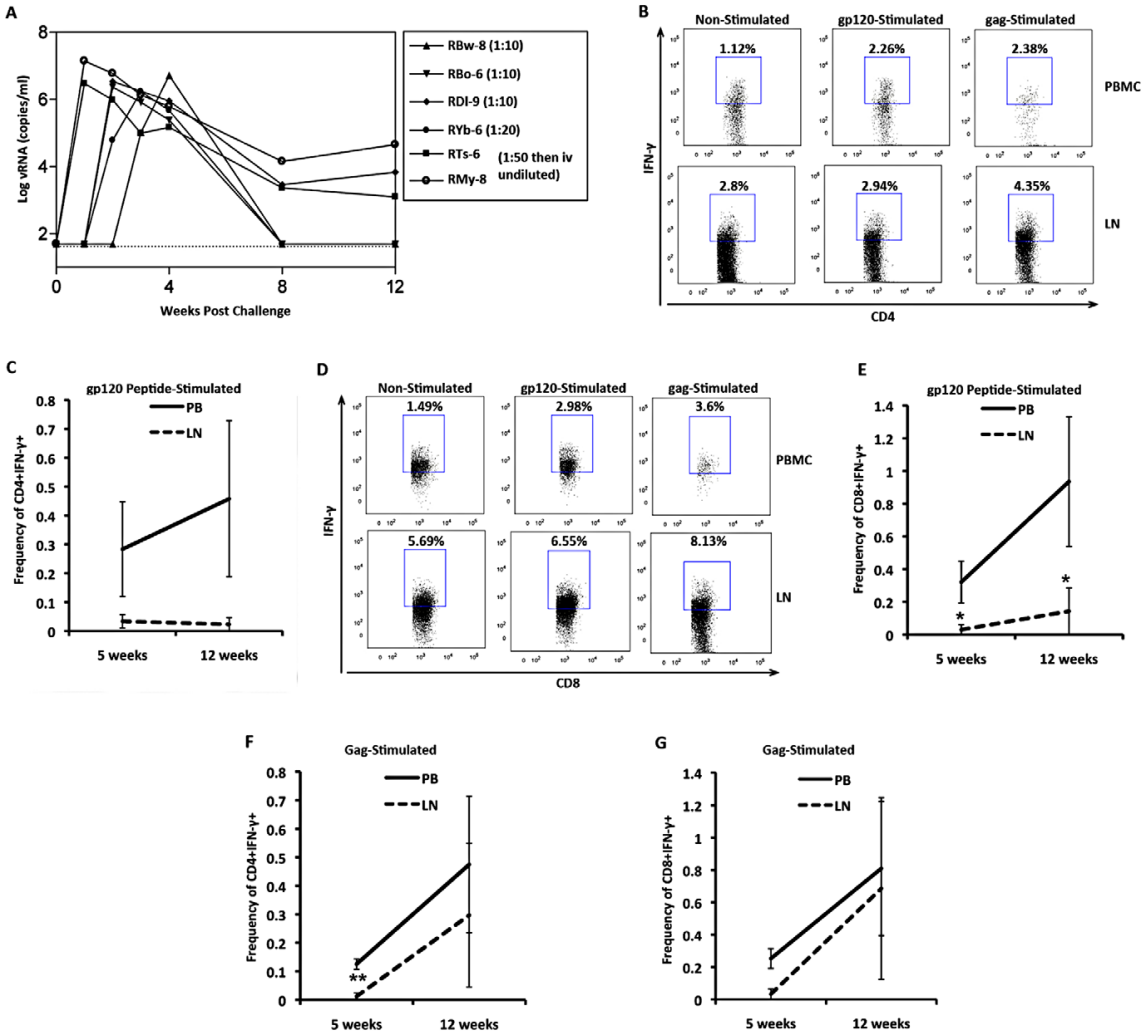
Reduced antigen-specific CD4 and CD8 T cell responses in LN RM during early R5-SHIV infection. RM were inoculated intravaginally (n = 4) or intravenously (n = 2) with SHIV-1157ipd3N4 (R5-SHIV). Blood samples were drawn for viral load analysis at 0, 1, 2, 3, 8 and 12 weeks post infection (A). Paired peripheral blood samples (PB) and lymph nodes (LN) from RM during early infection were sampled at 5 and 12 weeks post-inoculation (B-G). PB and LN lymphocytes were stimulated with overlapping clade C gp120 peptide pools at 5 and 12 weeks post inoculation and IFN-γ^+^ gp120-specific CD4 (B,C) and CD8 (D,E) T cell were assayed. F & G) PB and LN derived cells were stimulated with overlapping SIVmac239 Gag peptide pools at 5 and 12 weeks post inoculation and IFN-γ^+^ Gag-specific CD4 (F) and CD8 (G) T cell responses were assayed. Data representative of frequency of total (C,E-G) or frequency of parent (B,D) are shown. Error bars ±SEM, *p<0.05, ** p<0.01.

We examined responses of T cells derived from PB and LN upon stimulation with overlapping peptide pools of gp120 and Gag antigens at both 5 and 12 weeks post-inoculation. All animals were productively infected and had measurable viral loads at 5 weeks post inoculation. Half (3/6) of the RM subsequently had viral loads that were below the level of detection at 12 weeks post inoculation (Figure 1A). HIV-1gp120 specific IFN-γ+ CD4 T cells were lower in LN compared to PB, although this was not significant (Figure 1B &1C). However, the frequency of gp120-specific IFN-γ^+^ CD8 T cells was lower in LN than in PB at both 5 and 12 weeks post-inoculation (CD8 p<0.05, Figure 1D & 1E). Furthermore, in general gp120-specific CD4 and CD8 IFN-γ+ T cell responses in LN did not increase over time post R5-SHIV inoculation, whereas anti-gp120 CD4 and CD8 IFN-γ+ T cell responses in the PB increased over time between 5 and 12 weeks post-inoculation(Figure 1C & E). We also observed a different temporal pattern of Gag-specific responses. We observed a significant reduction with respect to Gag-specific CD4 T cells in LN as compared to PB at 5 weeks but not at 12 weeks post R5-SHIV inoculation (5 weeks: p<0.01, Figure 1F). Gag-specific CD4 and CD8 T cells increased over time regardless of anatomic location (Figure 1F & G). These data suggest that fewer gp120-specific T cells reside or are maintained in LN than in PB and that this effect is stable over time.

### Increased levels of Activation-Induced Cell Death (AICD) occur during early infection but are not associated with HIV-1 gp120 in lymph nodes

**Figure 2:**
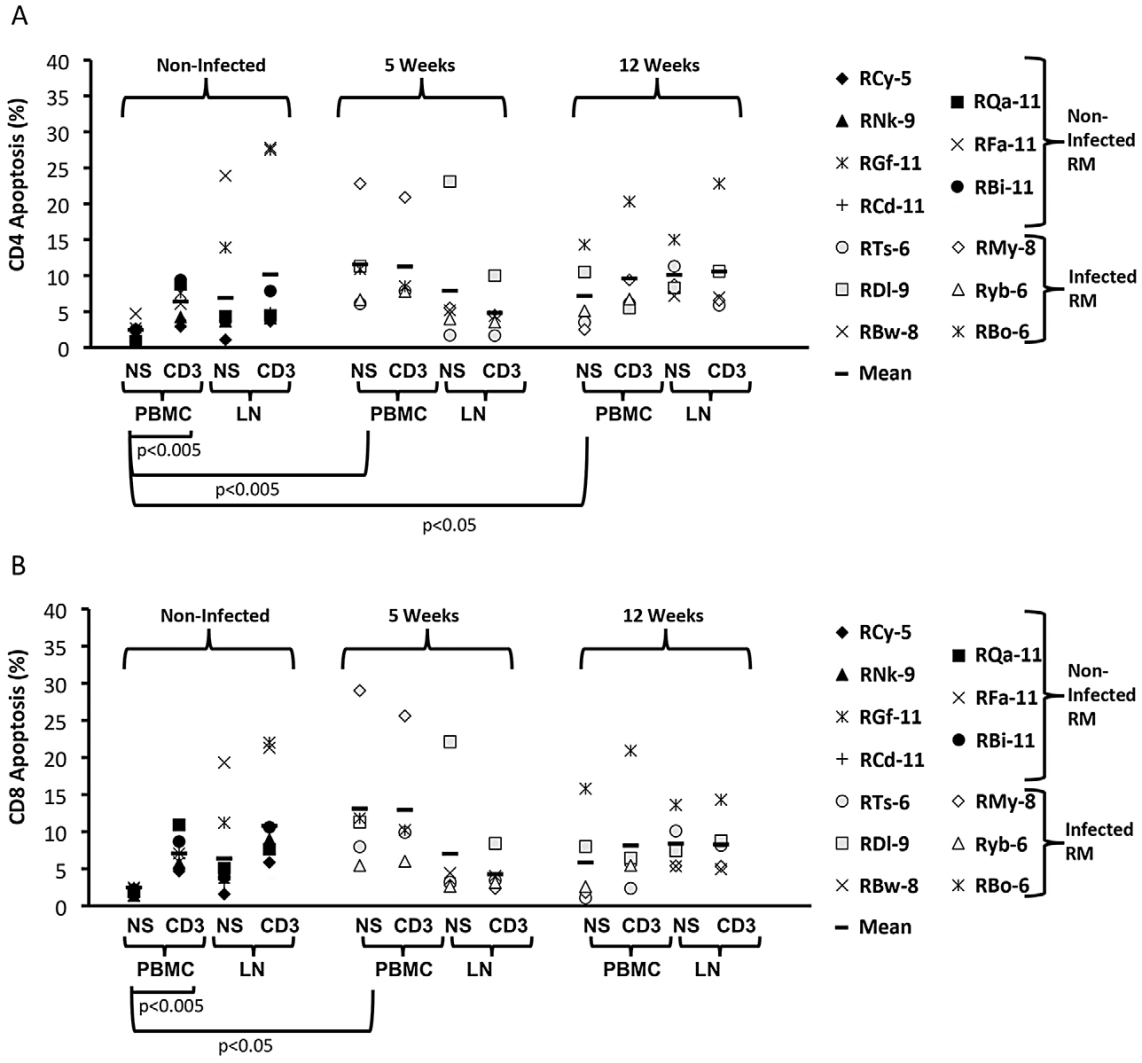
Enhanced basal T cell apoptosis from PB, but not LN of R5-SHIV-infected RM. Naïve and R5-SHIV challenged RM were sampled at 5 and 12 weeks post-inoculation. PB and LN samples from either naïve or 5 and 12 weeks post-inoculation samples were either non-stimulated (NS) or stimulated with plate-bound anti-CD3 (CD3) for 24 hours. A) Percent of apoptotic CD4 T cells from naïve RM or infected RM at 5 or 12 weeks post inoculation comparing non-stimulated to CD3 stimulated is shown (naïve PBMC non-stimulated vs naïve PBMC stimulated p<0.005; naïve PBMC non-stimulated vs 5 weeks infected PBMC non-stimulated p<0.005; naïve PBMC non-stimulated vs 12 weeks infected non-stimulated p<0.05). B) Percent of apoptotic T cells from naïve RM or infected RM at 5 or 12 weeks post inoculation comparing non-stimulated to CD3 stimulated, (naïve PBMC non-stimulated vs naïve PBMC stimulated p<0.005; naïve PBMC, non-stimulated vs 5 weeks infected PBMC, non-stimulated p<0.05).

Previous *in vitro* studies have demonstrated that gp120 can enhance T cell sensitivity to AICD and therefore we hypothesized that the relative depletion of anti-gp120 responses might be due to enhanced activation of LN-derived T cells [16], [20], [21], [37]. We examined the ability of T cells to undergo AICD after polyclonal stimulation and compared the results between T cells derived from either PB or LN from naïve RM or RM during early infection at 5 and 12 weeks post-inoculation. Upon examination of CD4 and CD8 T cells derived from naive animals, we found that PB-derived T cells demonstrated induction of AICD and stimulation-increased apoptosis, as expected (p<0.005 PBMC, Figure 2A & B). Interestingly, the basal levels of CD4 T cell apoptosis at 5 and 12 weeks (non-stimulated) were higher in infected RM as compared to PB derived T cells from naïve animals (p<0.005 PBMC 5 weeks, p<0.05 PBMC 12 weeks, Figure 2A). In contrast to naïve animals, PB-derived CD4 T cells from R5-SHIV-infected animals could not be further stimulated via anti-CD3 at either time point (Figure 2A). A similar pattern was observed with respect to CD8 T cells: PB-derived cells had higher basal apoptosis at 5 weeks post-inoculation compared to PB-derived T cells from naïve animals (p<0.05 PBMC naïve vs 5 weeks, Figure 2B). Moreover, LN-derived CD8 T cells did not demonstrate enhanced levels of apoptosis upon stimulation with CD3 at either 5 or 12 weeks post-inoculation (Figure 2B). Increased levels of AICD in LN were not correlated with HIV gp120 levels in these tissues (data not shown). These results suggest that AICD may not play a major role in immune dysregulation observed in LN of RM during early infection.

### Enhanced expression of PD-1 in LN derived T cells from RM during early infection

**Figure 3:**
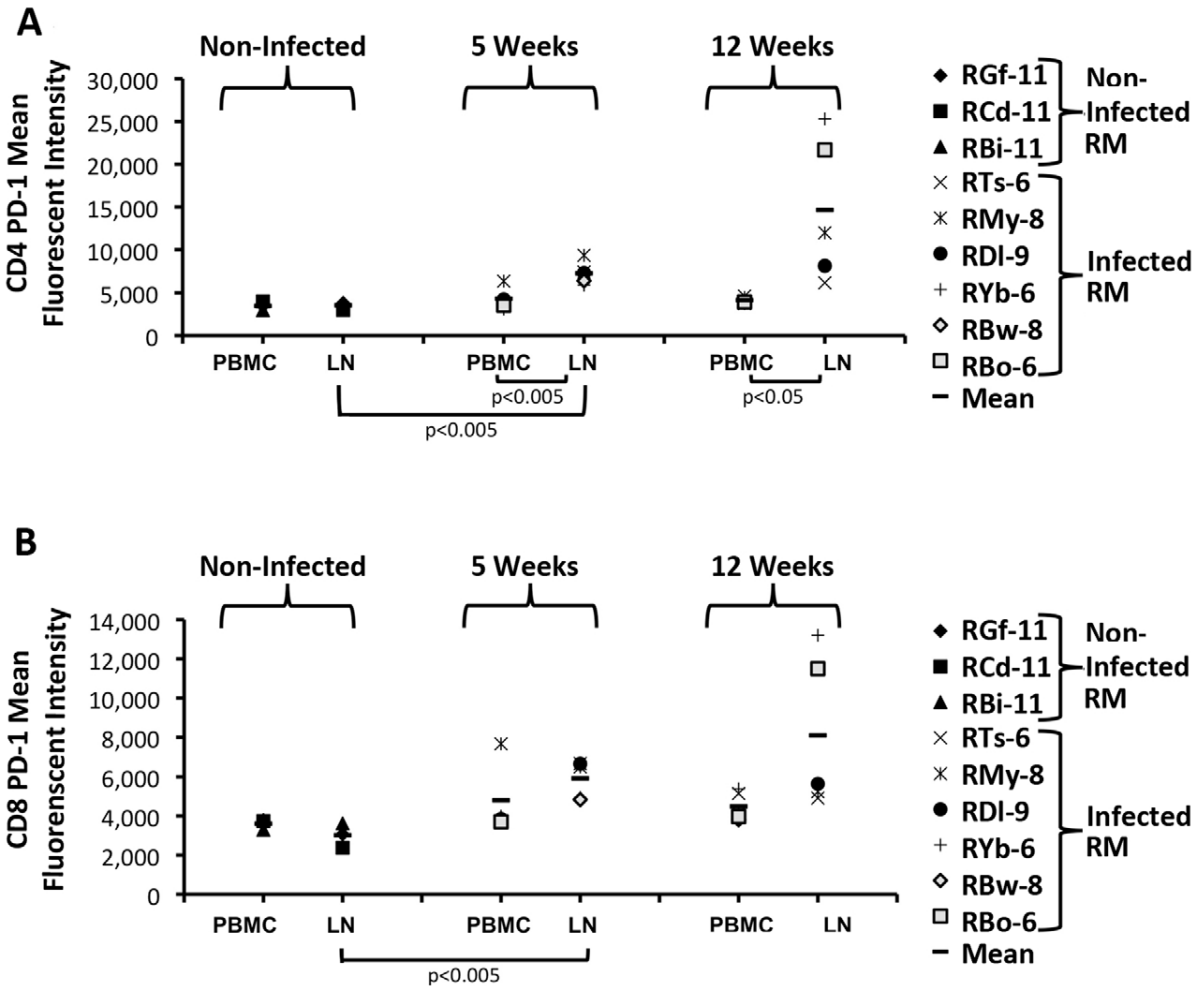
Enhanced expression of PD-1 on LN-derived CD4 and CD8 T cells. Naïve and intravaginally challenged RM were sampled at 5 and 12 weeks post-inoculation. Samples from PB and LN derived lymphocytes were examined by flow cytometry for the amount of PD-1 that was expressed at 5 and 12 weeks post-inoculation. Mean Fluorescent Intensity (MFI) for CD4 (A) and CD8 (B) of samples from infected and naïve RM are shown. Significant increases in PD-1 expression by LN derived CD4 and CD8 T cells were seen at 5 weeks post inoculation compared to naïve animals.

Although, we observed no differences in apoptosis that were associated with reduced antigen-specific LN responses we proposed that T cell anergy may in part account for the observed immune dysregulation. Previous studies have demonstrated that during chronic HIV infection, levels of PD-1 are upregulated and correlated with anergic T cell responses to HIV antigens [38], [39], [40]. Furthermore, the blockade of PD-1 restores T cell function in animal models [41], [42]. To further elucidate immune dysregulation in LN during early infection, we examined the level of PD-1 on T cells derived from PB and LN of naïve and infected RM at 5 and 12 weeks post-R5-SHIV inoculation. We observed no major differences with respect to the proportion of T cells expressing PD-1 in any of the groups (data not shown). However, when we examined the amount of receptor on a per-cell basis, we observed higher levels of PD-1 on LN-derived CD4 T cells from R5-SHIV-infected RM at 5 weeks compared to cells from naïve RM as measured by mean fluorescence intensity (MFI, p<0.005 Figure 3A). Whereas, PB-derived CD4 T cells at 5 weeks post-inoculation had levels of PD-1 similar to those seen in naïve animals (Figure 3A). In general, we also observed more PD-1 on CD4 T cells derived from LN at both 5 and 12 weeks post-inoculation as compared to PB-derived CD4 T cells (5 weeks p>0.005,12 weeks p<0.05 Figure 3A). We also observed a similar phenomenon with respect to LN-derived CD8 T cells, where PD-1 receptor expression was upregulated at 5 weeks post-inoculation compared to cells from naïve RM (p<0.005, Figure 3B). These data suggest that upregulation of PD-1 can be observed in LN-derived CD4 T cells of R5-SHIV challenged RM during early infection in LN and not in PB.

### Increased frequency of CD4^+^CD25^+^CD127^low^ T cells in LN of RM during early infection correlate with tissue levels of gp120

**Figure 4:**
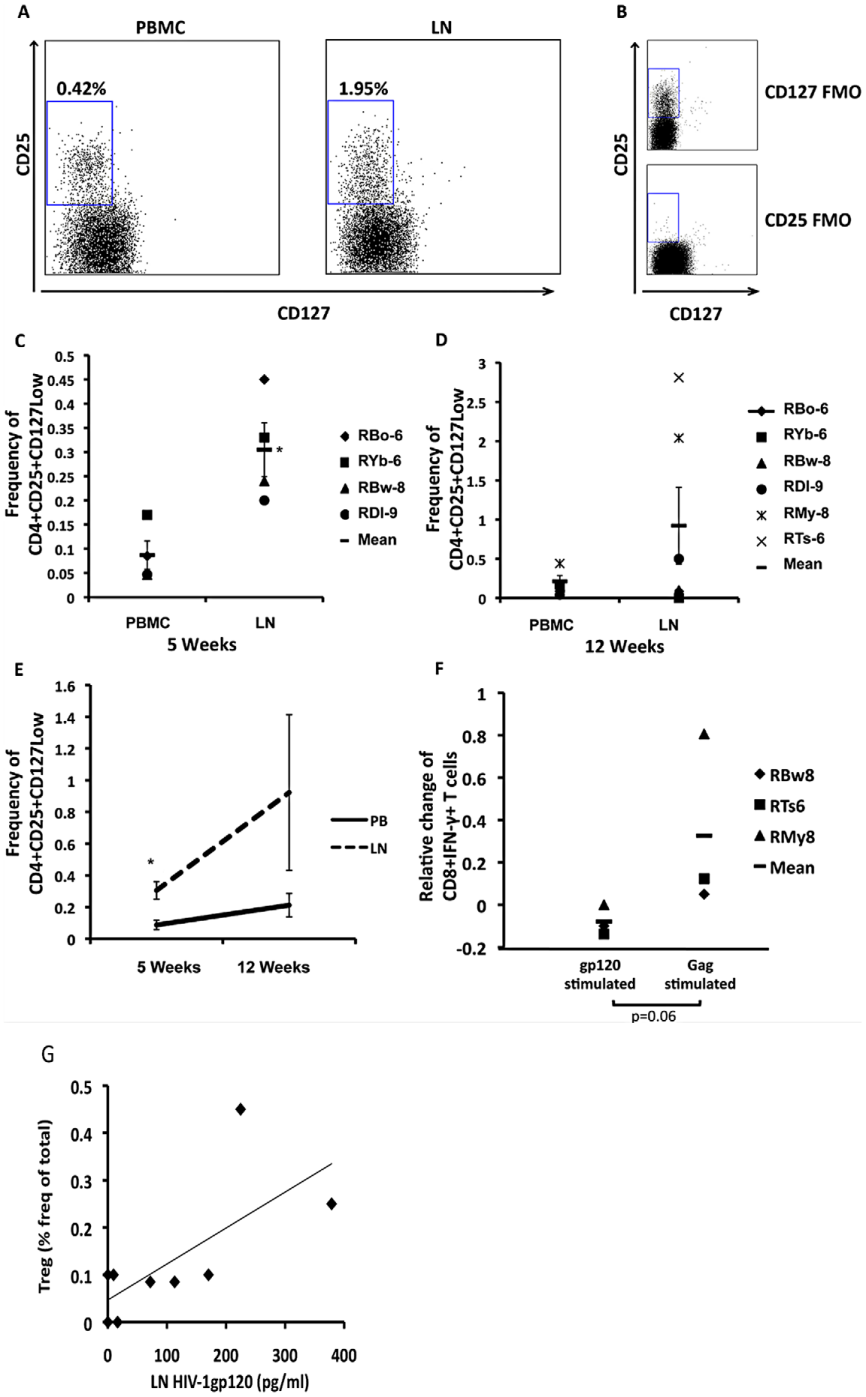
Increased numbers of CD4+CD25+CD127low T cells in the LN of R5-SHIV infected RM. RM were sampled at 5 and 12 weeks post-inoculation. PB and LN samples were examined by multi-colour flow cytometry for the proportion of Treg cells present. A) Representative dotplots from PB and LN gated on size and granularity as well as CD3 and CD4, and subsequently on CD25 vs CD127 as shown (% frequency of parent). B) Gating strategy demonstrating the fluorescent minus one scheme utilized as above and CD25 vs CD127 as shown. C & D) Individual RM Treg cell frequencies of parental gate at 5 weeks post-infection *p<0.05 LN compared to PB (C), and 12 weeks (non-significant) (D) post-infection. E) Time course of Treg accumulation at 5 and 12 weeks post infection in PB and LN, mean ±SEM. F) LN samples depleted of CD4+CD25+ T cells and stimulated with overlapping SIVmac239 Gag peptide pools. Data depicted as relative change of Gag- and gp120 specific CD8 responses in CD4+CD25+ depleted compared to non-depleted samples, (p = 0.06 vs change in gp120 specific CD8 T cells.) G) Correlation of Tregs with gp120 in the LN of intravaginally challenged RM (r^2^ = 0.7, two-tailed p = 0.035).

We hypothesized that lower frequencies of gp120-specific CD4 and CD8 T cells and to some extent Gag-specific CD4 and CD8 T cells seen in LN compared to PB, resulted from antigen-specific Treg suppression. To address this, we examined the proportion of CD4^+^CD25^+^CD127^low^ Treg in PB and LN of SHIV challenged RM during early infection. We observed CD4^+^CD25^+^CD127^low^ T cells in both PB and LN of RM during early infection (Figure 4A & B). We found consistently higher frequencies of CD4^+^CD25^+^CD127^low^ T cells at 5 weeks post-inoculation in LN compared to PB (>85% of all CD4^+^CD25^+^CD127^low^ T cells were FoxP3^+^) (5 weeks: p<0.01, Figure 4C, E). In order to determine whether the observation of increased frequencies of CD4^+^CD25^+^CD127^low^ T cells was associated with decreased frequencies of IFN-γ^+^ T cells in LN, we examined the effect of Tregs on antigen-specific T cell production of IFN-γ. To accomplish this, we depleted 5 week post-inoculation PB and LN mononuclear cell samples of CD4^+^CD25^+^ cells, where we observed significant differences in the frequencies of Treg and the greatest difference between IFN-γ producing, antigen-specific T cells (gp120 and Gag) from PB and LN. CD4^+^CD25^+^ depletion reduced the level of this subpopulation by 78±8%. We observed a trend toward more Gag-stimulated CD8 T cells producing IFN-γ in the CD4^+^CD25^+^-depleted samples than in the non-depleted samples, as shown by the difference between depleted compared to non-depleted cells (p = 0.06, Figure 4F). Interestingly, gp120-stimulated CD8 T cells were not impacted by the depletion of CD4^+^CD25^+^ T cells (data not shown), which is similar to observations in mouse models where the effects of Tregs are most potent on immunodominant epitopes [43], [44], [45]. Although Treg depletion had no impact on the restoration of CD4 T cell responses, these results may be difficult to interpret due to the fact that the Treg depletion step may have reduced the proportion of effector T cells in our samples. Finally, we observed that increasing levels of CD4^+^CD25^+^CD127^low^ T cells in LN of RM at 5 weeks and 12 weeks post-inoculation positively correlated with the measured concentration of gp120 in lymphoid tissues (Figure 4G, r^2^ = 0.7 p = 0.035).

### Tregs migrate towards R5 gp120 in a CCR5 and G-protein coupled receptor manner

**Figure 5:**
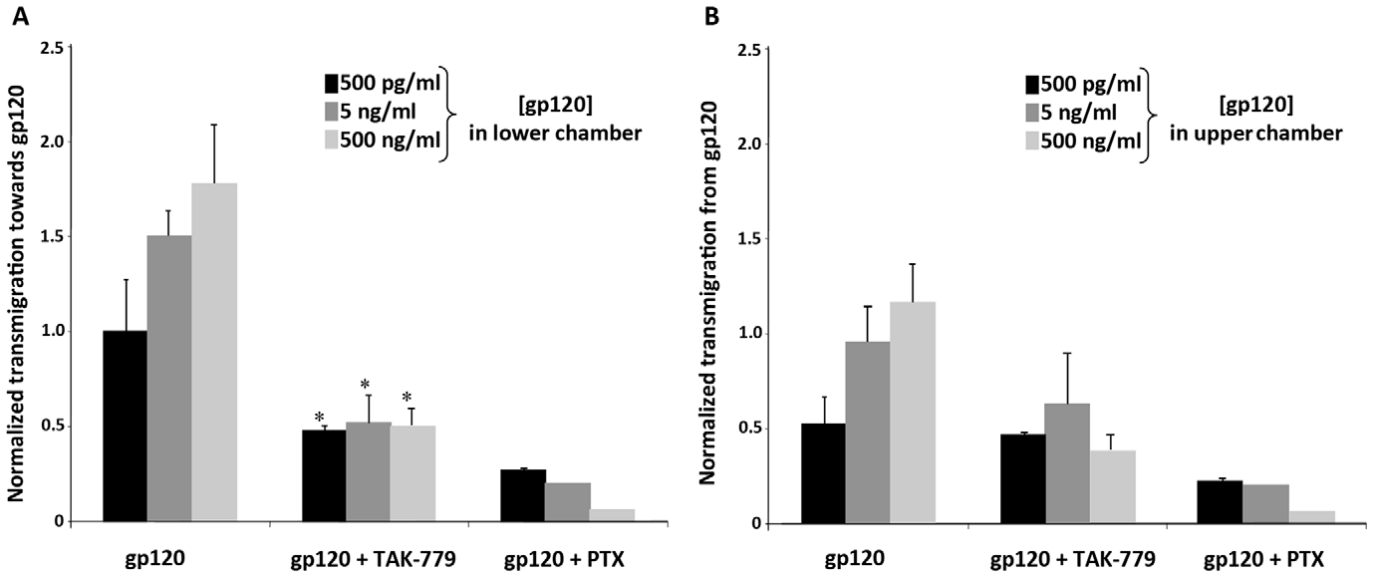
T regulatory cells migrate toward R5 HIV gp120. CD4+CD25+T regulatory cells were purified from naive human PBMC and exposed to various concentrations of gp120 in a Boyden chamber migration assay. A) Normalized transmigration index of Tregs that migrate towards various gp120 concentrations (500 pg/ml, 5 ng/ml, 500 ng/ml) in presence of CCR5 antagonist, TAK-779, or pretreated with pertussis toxin (Ptx 100 ng/ml) (*p<0.05 vs TAK-779). B) Normalized transmigration index of Tregs that migrate away from gp120 concentrations (500 pg/ml, 5 ng/ml, 500 ng/ml) in presence of CCR5 antagonist, TAK-779 (40 nM), or pretreated with pertussis toxin (Ptx, 100 ng/ml).

In view of the finding that LN levels of gp120 correlated with the number of Tregs in this tissue, we hypothesized that gp120 may play a direct role in Treg recruitment. To examine this, we investigated the migration of Tregs from human HIV naïve donors in response to recombinant R5 HIV-1 gp120. We observed that Tregs migrated with increasing frequency to an R5 (YU2) gp120 in a dose-dependent manner (Figure 5A). Furthermore, the specific CCR5 antagonist, TAK-779, inhibited Treg migration demonstrating the dependence of this directional migration on CCR5. Additionally, pertussis toxin, an inhibitor of G-protein coupled receptor signaling, potently inhibited Treg migration toward R5 gp120 (Figure 5A). Finally, we observed that Tregs do not migrate away from R5 gp120 at any concentration of envelope protein that we examined (Figure 5B). These results suggest that recruitment of Tregs to lymphoid tissues during HIV infection may be in part due to the chemoattractant activity of R5 gp120 for this T cell subpopulation.

## Discussion

In this study, we demonstrate a significant impact of early R5-SHIV infection on critical aspects of T cell function at and beyond the 5-week time point of maximal CD4 T cell depletion and viral load, including upregulation of PD-1 expression, differential suppression of gp120-specific T cell responses and preferential accumulation of Treg cells in lymphoid tissues. These immune dysregulatory effects are associated with high levels of the envelope protein (gp120) of HIV in lymphoid tissues when viral load is low or undetectable in PB (12 weeks). These data are consistent with a growing literature describing the effects of gp120 on T and B-cell function *in vitro* [10], [11], [12], [13], [14], [15], [16], [17], [19], [23] and gp120-mediated dysregulation of immune cell function and localization *in vivo* [22], [27], [29], [46], [47].

Our results reveal consistent differences between the measurements of immune activation and regulation of PB versus LN-derived CD4 and CD8 T cells, regardless of route of infection (intravenous or mucosal). These differences are also evident despite the small sample size and the inclusion of two animals in this study that were challenged via a non-mucosal route and are consistent with a similar study performed in the dual-tropic SHIV-KB9 model using intravenous transmission [27]. In addition, these data imply that the anti-HIV immune response during early infection could easily be overestimated if the responses generated by circulating T cells was the only measurement made in this context. The examination of PB T cell function as a surrogate marker of immune activation in lymphoid tissues in HIV has been the gold standard in both basic science and clinical trials. Interestingly, there is an increasingly consistent lack of correlation between PB T cell responses and immune protection in RM models [24], [27], [48], [49]. Although immune cells residing in LN are more difficult to sample, they may provide a deeper understanding of the mechanisms used by the virus to subvert and evade host immune responses.

The presence of gp120 in LN of RM during early infection was shown to be associated with dysregulated IFN-γ responses of CD4 and CD8 T cells [27]. Previously, our laboratory demonstrated that the addition of exogenous gp120 to PB CD4 and CD8 T cells reduced HIV-specific IFN-γ responses to those levels observed in LN [27]. In the current study, increased levels of gp120 and the impaired IFN-γ response observed in LN were associated with increased levels of the T cell exhaustion marker, PD-1. Moreover, we observed enhanced basal apoptosis in PB of infected RM, suggesting that apoptosis may play a role in immune dysregulation, but this did not correlate with LN gp120 levels, PD-1 levels or impaired IFN-γ responses. The lack of correlation to apoptosis may be due to the fact that tissues have differential apoptotic rates during early infection [50]. Although HIV gp120 has been demonstrated to upregulate T cell death *in vitro*, we were unable to find a direct correlation to this parameter *in vivo* in this model [16], [19], [21].

Regulatory T cells have been demonstrated to have either a deleterious effect or no effect on HIV infection [51], [52], [53], [54], [55]. Here, we show that increased numbers of CD4^+^CD25^+^CD127^low^ Treg correlate to the levels of gp120 in the LN at 12 weeks post infection and that the LN resident Tregs are in part responsible for the dampening of ex vivo CD8 IFN-γ responses against Gag antigen. Treg depletion resulted in a trend toward higher Gag-specific T cell responses but did not enhance CD8 gp120-specific T cell responses. This finding carries the caveat that it is difficult to interpret the effect of Treg depletion on Gag and gp120-specific CD4 T cells as we deplete a population of CD4^+^CD25^+^ that may include T effector/memory cells [56]. These results support previous findings in mouse models, where immunodominant epitopes are preferentially targeted by Tregs [43], [44], [45]. Our data also suggest that other mechanisms, such as direct suppression via gp120, may play a role in immune dysregulation. *In vitro* studies suggest that gp120 itself can suppress the immune response independent of Treg [57]. Interestingly, a recent study by Becker et al. reported that a single dose of HIV-1 gp120 was able to ameliorate graft versus host disease in a mouse via the specific activation of Tregs [58]. Our *in vitro* data demonstrate that Tregs migrate toward R5 gp120 in a CCR5/Gαi-protein coupled receptor-dependent manner. We propose that the accumulation of Tregs in lymphoid tissue during acute R5-SHIV infection may be completely or partially driven by HIV-1 gp120 induced Treg cell chemoattraction.

This study is the first demonstration of multimodal dysregulation of T cell function that occurs *in vivo* during early mucosal challenge with R5-SHIV. Furthermore, these data support the view that the persistence of HIV-1 gp120 in lymphoid tissues during early infection is associated with dysregulation of T cell function beyond CD4 T cell depletion that is emblematic of HIV/AIDS. Further examination of the effects of the virus and its envelope protein on HIV-1 antigen specific responses in lymphoid tissues *in vivo* at early time points following virus inoculation will assist in the broader understanding of the pathogenesis of HIV infection and those aspects of the disease which will need to be prevented or reversed by a vaccine or viral eradication approach.
